# Spatial distribution and trypanosome infection of tsetse flies in the sleeping sickness focus of Zimbabwe in Hurungwe District

**DOI:** 10.1186/s13071-016-1879-5

**Published:** 2016-11-25

**Authors:** William Shereni, Neil E. Anderson, Learnmore Nyakupinda, Giuliano Cecchi

**Affiliations:** 1 Tsetse Control Division, Department of Livestock and Veterinary Services, Ministry of Agriculture, Mechanization and Irrigation Development, Harare, Zimbabwe; 2 The Royal (Dick) School of Veterinary Studies and the Roslin Institute, University of Edinburgh, Easter Bush Campus, Roslin, UK; 3 Food and Agriculture Organization of the United Nations, Sub-Regional Office for Eastern Africa, Addis Ababa, Ethiopia

**Keywords:** Tsetse, *Glossina*, Sleeping sickness, African animal trypanosomiasis, Zimbabwe, Epidemiology

## Abstract

**Background:**

In Zimbabwe, cases of human African trypanosomiasis (HAT) are caused by the unicellular protozoan *Trypanosoma brucei*, sub-species *T. b. rhodesiense*. They are reported from the tsetse-infested area in the northern part of the country, broadly corresponding to the valley of the Zambezi River. Tsetse-transmitted trypanosomes, in particular *T. congolense* and *T. vivax*, also cause morbidity and mortality in livestock, thus generating poverty and food insecurity. Two species of tsetse fly, *Glossina morsistans morsitans* and *G. pallidipes*, are known to be present in the Zambezi Valley, although their distributional patterns and densities have not been investigated in detail. The present study tries to address this gap by providing some insight into the dynamics of trypanosomiasis in humans and livestock.

**Methods:**

Tsetse distribution and trypanosome infections were studied using traps and fixed fly rounds located at 10 km intervals along a 110 km long transect straddling the southern escarpment of the Zambezi Valley. Three km long fly rounds were conducted on 12 sites, and were repeated 11 times over a 7-month period. Additional traps were deployed and monitored in selected sites. Microscopic examination of 2092 flies for trypanosome infections was conducted.

**Results:**

Surveys confirmed the presence of *G. morsitans morsitans* and *G. pallidipes* in the Zambezi Valley floor. Moving south, the apparent density of tsetse flies appears to peak in the vicinity of the escarpment, then drops on the highlands. Only one fly was caught south of the old game fence separating protected and settled areas. A trypanosome infection rate of 6.31% was recorded in tsetse flies dissected. Only one infection of the *T. brucei*-type was detected.

**Conclusions:**

Tsetse fly distribution in the study area appears to be driven by ecological factors such as variation in land use and altitude-mediated climatic patterns. Although targeted control of tsetse flies have played a role in determining distribution, no major control operations have been implemented in the area for 15 years. Trypanosome infections in tsetse flies are consistent with HAT epidemiological data, which considers the situation to be generally ‘low risk’. Nonetheless, underreporting is likely to conceal the true epidemiological picture, and efforts are needed to strengthen the diagnostic capacities of health facilities.

## Background

Tsetse flies (Diptera: Glossinidae) are the vectors of both human and animal trypanosomiasis, which continue to impose a heavy burden on human and livestock health in sub-Saharan Africa [1]. Thirty-one species and subspecies of tsetse flies inhabit approximately 11 million km^2^ between 15°N and 29°S [2, 3]. Of the 31 species, two of the savannah group are found in Zimbabwe: *Glossina morsitans morsitans* and *Glossina pallidipes*. Half the area of Zimbabwe below 1000 m altitude (approximately 200,000 km^2^) is considered ecologically suitable for tsetse flies and therefore it was historically unsuitable for livestock rearing. Following the rinderpest epidemic in 1896, tsetse flies disappeared from the banks of the Zambezi River between Victoria Falls and the Gwayi River [4, 5]. Furthermore, subsequent tsetse control operations using a combination of techniques such as ground spraying, aerial spraying and bait-technologies (insecticide-treated cattle and targets), reduced the area of tsetse habitat to the present level (approximately 30,000 km^2^). The tsetse flies distribution in Zimbabwe is presently mainly restricted to the northern districts along the Zambezi Valley.

In our study area, (Hurungwe District, northern Zimbabwe), major tsetse control operations using insecticide-treated and odour-baited targets were discontinued in 2001. Since then, no major control activities have been implemented in the area south of the game fence, separating protected and settled areas except cattle dipping with deltamethrin. The formulation used is effective in controlling ticks as well as tsetse flies. Dipping of cattle is conducted fortnightly during the dry season (May to October) and weekly during the rainy season (November to April). Cases of trypanosomiasis in livestock are diagnosed at dip tanks during regular monitoring surveys.

The present study sought to explore the spatial distribution and trypanosome infection rates of tsetse flies in the human African trypanosomiasis (HAT) endemic area of Zimbabwe (Hurungwe district). In parts of this area the tsetse fly habitat has been modified through deforestation and the expansion of agriculture. These changes necessitated reassessment of tsetse fly distribution patterns in the area. At the national level, the Tsetse Control Division in Zimbabwe has recently embarked on the development of a National Atlas of tsetse flies and African animal trypanosomiasis (AAT), to which the present study will contribute. The Atlas will broadly follow the methodologies developed by the Food and Agriculture Organization of the United Nations (FAO) for the continental Atlas of tsetse flies and AAT [6, 7]. It will also draw upon the example of other national-level Atlases, such as the one recently developed in Sudan [8].

The present study was also necessitated by the upsurge in HAT cases that were reported in 2012 [9], and the need to explore its causes. No case of HAT was reported in Zimbabwe between 1998 and 2004 [9]. By contrast, 28 cases and three deaths were recorded between 2005 and 2015, all originating from the Hurungwe District and neighbouring areas. Little is known of the causes of the observed resurgence of the disease in the past decade. Reported cases include local inhabitants and foreign travellers [10, 11], together with cases reported from neighbouring areas in Zambia [10]. Many infections in Zimbabwe were detected in tourists who visited the Zambezi Valley, as well as in game park rangers who spend much of their time in tsetse fly habitat. Furthermore, limited diagnostic capacity for HAT in the national health system is likely to result in important levels of under detection, thus distorting the true epidemiological situation.

## Methods

### Study area

**Fig. 1:**
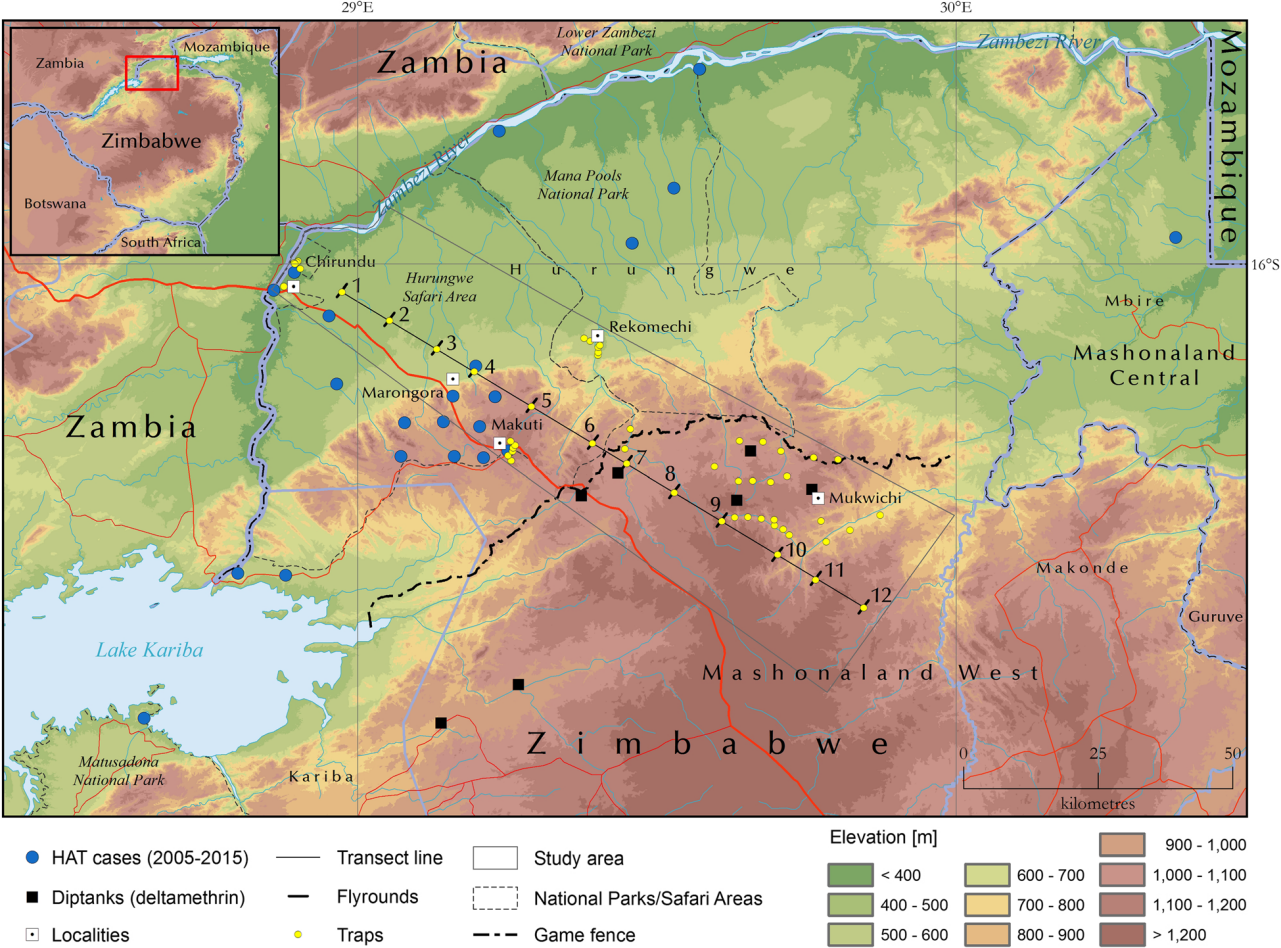
The area in Zimbabwe (Zambezi escarpment, Hurungwe District) that was the focus of this study. The source of human African trypanosomiasis cases is the WHO Atlas of HAT, Simarro et al. [10]. *Abbreviation*: HAT, human African trypanosomiasis

The surveys presented in this paper were conducted in the Hurungwe District (Mashonaland West Province, northern Zimbabwe; Fig. 1). In particular, a 110 km long transect was investigated, intersecting the southern escarpment of the Zambezi Valley. The lowlands north of the escarpment are protected areas and are home to a rich variety of wild animals, including elephant, buffalo, impala, warthog and cheetah. In this environment, wildlife provides the main source of blood meals for tsetse flies. The main tree species are *Colophospermum mopani*, *Acacia nigrescens*, and *Adansonia digitata*. The area is also characterized by a dense undergrowth of deciduous bushes [12]. On the highlands south of the escarpment, land use is characterised by mixed crop agriculture, livestock and by higher human population densities. Until recently, a game fence physically marked the boundary between the protected and settled areas. This fence is no longer regularly maintained along its length, but still represents the administrative limit of the protected areas. As such, it continues to mark a sharp discontinuity in land use. The study was conducted over the four seasons (hot-dry, warm-wet, cool-wet, and cool-dry) in Zimbabwe.

### Design of tsetse surveys

#### Transect design

A transect from the floor of the Zambezi Valley (at an altitude of approximately 400 m), up the escarpment and ending on the highlands (at approximately 1200 m) was used for the survey. A random start point was selected for the first sampling location and an interval of 10 km was used for subsequent locations, providing a total of 12 sampling sites along the transect (Fig. 1). A combination of blue/black/blue-screen fly rounds [13, 14] and Epsilon traps were used for sampling.

#### Screen fly rounds

For each of the 12 sampling sites a 3 km long fly round perpendicular to the transect was carried out. Each fly round was divided into equal sectors of 100 m, which were marked on the ground with white paint. Geographical coordinates were systematically measured with the global positioning system (GPS). Fly rounds involved the use of a blue/black/blue screen attached to an aluminium pole that was baited with tsetse fly odour attractants (3-n propyl phenol, 1-octen-3-ol, 4-methyl phenol). The screen was carried by two men equipped with fly nets for catching all the flies landing on the screen [15]. The surveys were conducted simultaneously on all fly rounds for 5 h (from 08:00 to 10:30 h in the morning and from 14:00 to 16:30 h in the afternoon), taking into consideration the bimodal pattern of tsetse fly activity [16]. Some of our teams were working in National Park areas and limited transport prompted us to end the fly rounds at 16:30 h. This decision was mainly to allow teams to be retrieved from the park before it was dark to avoid encounters with wildlife after sunset. Eleven sessions, with each comprising two fly rounds, one conducted in the morning and the other in the afternoon of each sampling day, were simultaneously conducted at each of the 12 sites, starting on 26 February and ending on 18 November 2014. The study period covered both dry and rainy seasons.

#### Transect traps

In addition to fly rounds, stationary traps (Epsilon traps) were used to monitor tsetse flies along the transect. Two traps were deployed at each of the 12 sites where fly rounds were conducted. In particular, at each site one trap was located at the exact intersection between the transect line and the fly round line, while the second trap was deployed at a distance of 100 m from the intersection. Efforts were made to optimize trap visibility, by choosing more open sites where traps were not obscured by dense vegetation [17]. The 1 m radius clearings around the traps were intended to prevent damage by veld fires. All Epsilon traps were 90 cm tall and 1 m wide and consisted of phthalogen blue cloth with sections of black cloth inside the trap. The traps were baited with a 1:4:8 mixture of 3-n propyl phenol: 1-octen-3-ol: 4- methyl phenol [18]. Acetone was dispensed from 500 ml bottles with an aperture on the lid of 5 mm in diameter. Traps were maintained and monitored during the same period when fly rounds were conducted.

#### Additional traps

The results of fly rounds and trapping along the transect were complemented by additional tsetse fly sampling using Epsilon traps carried out in selected sites in the study area. Thirty-nine of these additional traps were located at sites belonging to a network of long term tsetse fly monitoring sites in the Hurungwe District, below and above the Zambezi escarpment. These traps were broadly clustered around five sites, namely Chirundu, Marongora, Makuti, Rukomechi and Mukwichi (Fig. 1). From this long-term (22 years) monitoring dataset, in this paper we present only the catches for a 2-year period (January 2014 to December 2015). In addition, eight additional traps were strategically deployed in suitable habitat in the area south the escarpment, where tsetse flies were expected to be either absent or present at very low densities based on local knowledge. These traps were monitored between December 2014 and March 2015. Apparent density was calculated as the number of flies captured in a trap as a fraction of the number of days the trap was operational.

### Tsetse infection

All 92 flies captured along the transect between April and June 2014 (either during fly rounds or in traps) were examined for trypanosome infection with microscopy according to Lloyd and Johnson [19]. Dissection was also performed on 2000 randomly selected flies collected in one of the sites on the valley floor (Rukomechi), where additional traps were deployed (April 2014 and December 2015).

### Statistical analysis

Statistical analyses were carried out using Minitab 17, R 3.1.2 and Statistica 8 software [20–22]. Analysis of variance (ANOVA) was conducted for both fly rounds and transect traps to assess whether the difference between means was statistically significant. Graphs for tsetse fly catches using both methods were generated to indicate the level of standard error. T-tests were conducted on morning and afternoon tsetse fly catches on fly rounds as well as on infection rates of tsetse flies captured along the transect compared to those at Rukomechi. Non-parametric tests (Wilcoxon rank sum test) were conducted on tsetse fly species caught in traps and on fly rounds due to the non-normal distribution of the data. Shapiro-Wilk normality test were conducted on data to test the hypothesis of data normality. Where the test indicated data normality, *t*-test was used and where it did not, the Wilcoxon rank sum test was used.

## Results

### Fly rounds

**Fig. 2:**
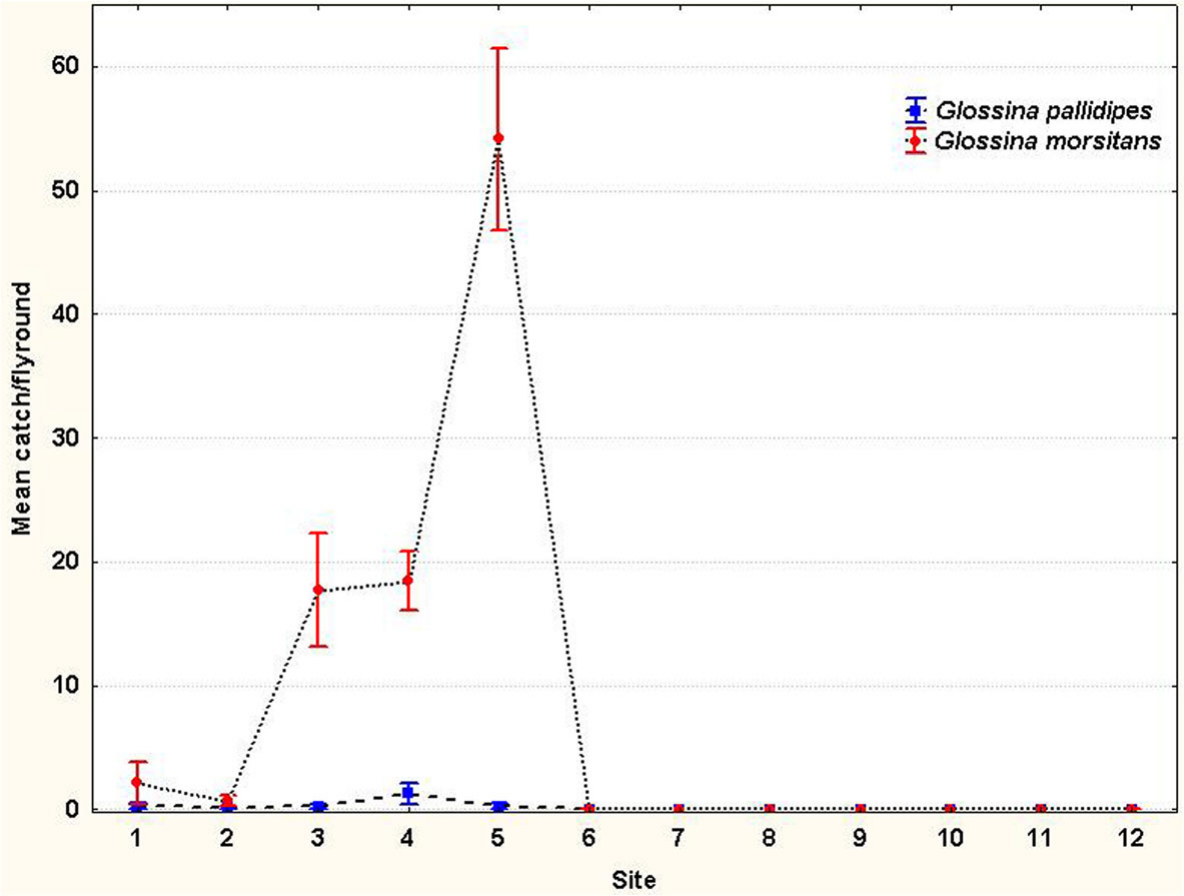
Mean tsetse catches along the study transect (fly rounds)

Entomological data from the 12 fly rounds revealed the presence of tsetse flies from the Zambezi Valley floor to the top of the escarpment (fly round site one to five), while tsetse flies were not caught on the highlands (fly round sites six to twelve). Tsetse flies were caught in the greatest numbers at the top of the escarpment (fly round 5, ANOVA: *F*_(11,108)_ = 12.51, *P* < 0.0001 ), with numbers progressively decreasing northward to the valley floor (Fig. 2). *Glossina m. morsitans* was the most abundantly caught tsetse fly in the fly rounds (*n* = 652), while considerably fewer *G. pallidipes* were caught (*n* = 16). The difference was strongly significant (0.1294 average catches for *G. m. morsitans* *vs* 0.0032 for *G. pallidipes* per flyround sector, ANOVA: *F*_(1,298)_ = 69.61, *P* < 0.001). Morning and afternoon catches showed no statistically significant difference (*t*-test: *t*_(12)_ = -0.04, *P* = 0.969). The Shapiro-Wilk normality test resulted in *P*-values of 0.3981 and 0.3877 for morning and afternoon catches, respectively. Values greater than 0.05 indicate that the hypothesis for data normality is accepted.

### Transect traps

**Fig. 3:**
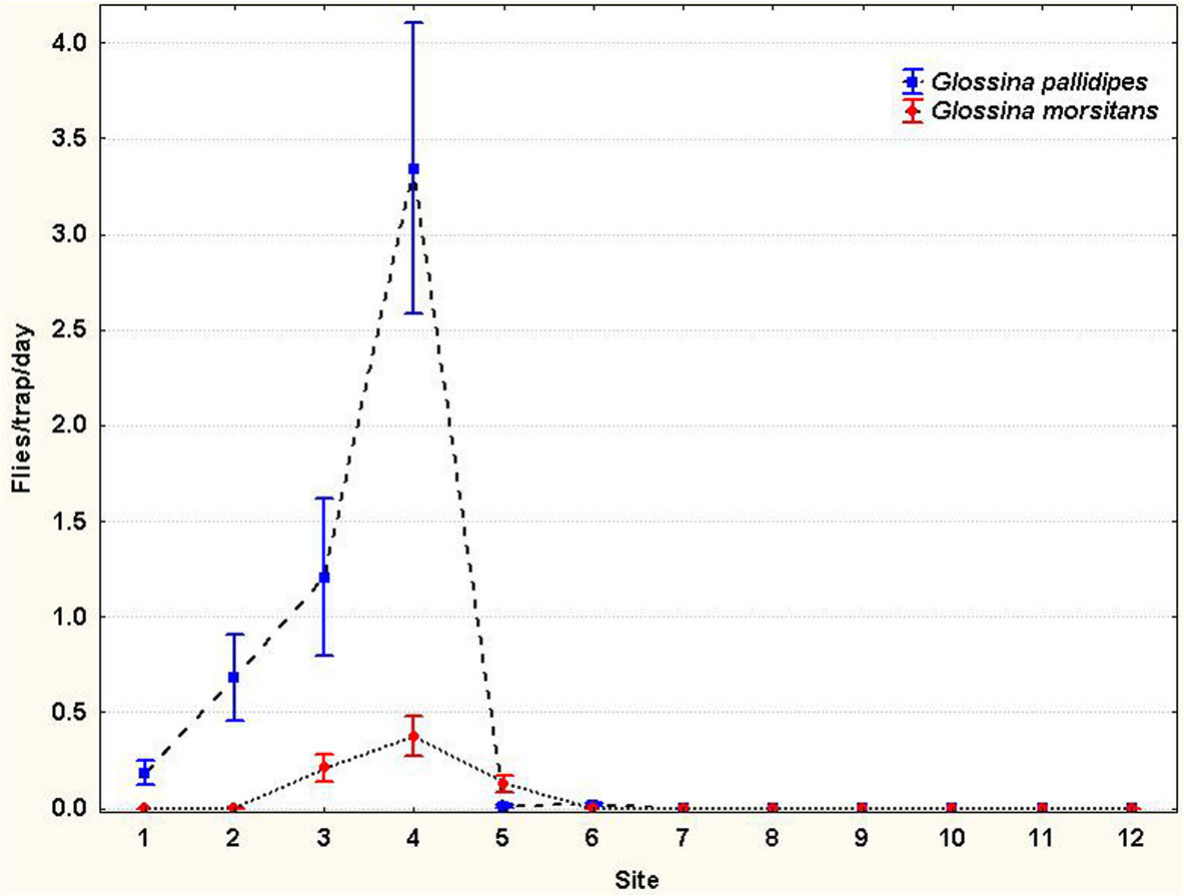
Tsetse apparent densities along the study transect (traps)

**Fig. 4:**
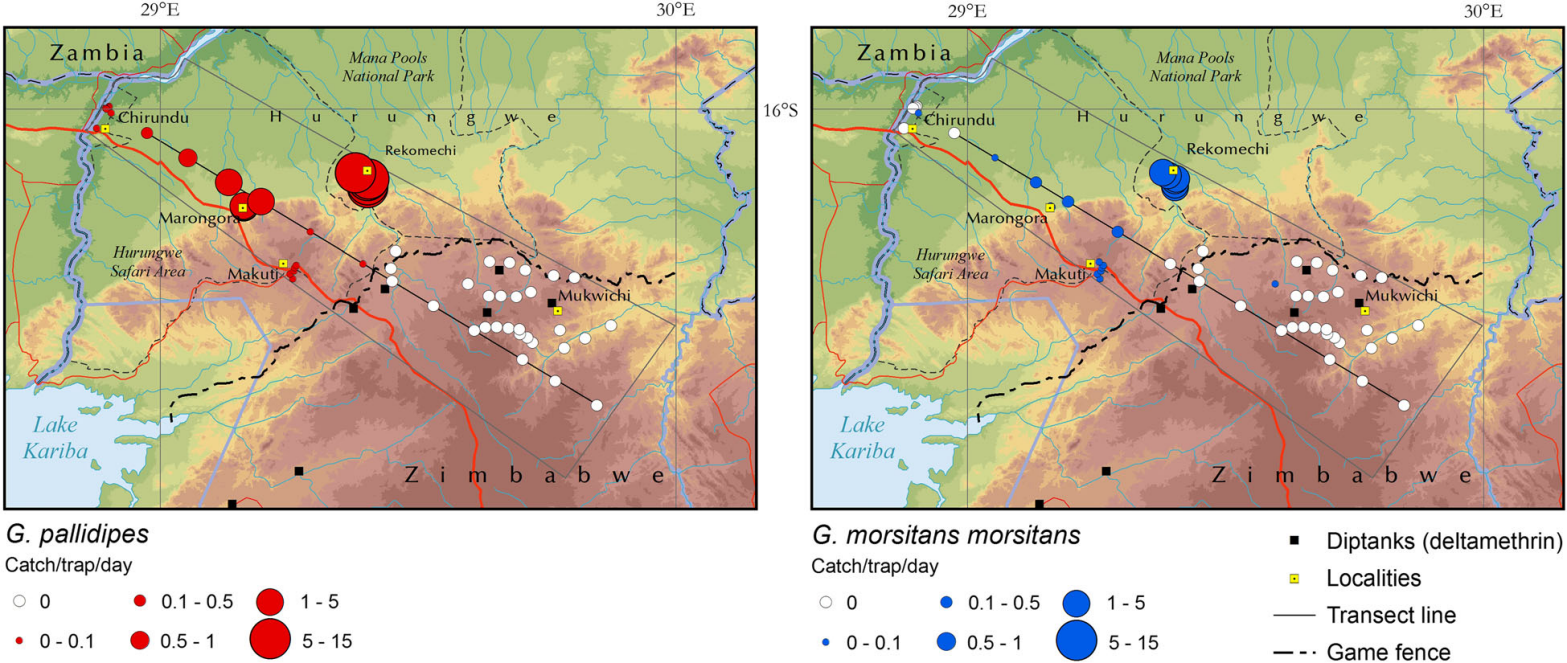
Tsetse apparent density (transect traps and additional traps)

Entomological data collected along the transect by means of stationary traps confirmed, to a large extent, the distributional pattern revealed by fly rounds (Fig. 3). The presence of tsetse flies was detected from the Zambezi Valley floor to the top of the escarpment (sites one to six), but not on the plateau (sites 7 to 12). The peak in tsetse fly density (i.e. 72 flies/trap/day) was observed at the foot of the escarpment (site 4). The results are also mapped in Fig. 4.

One notable difference between the results of stationary traps and fly rounds was the most abundant species caught: while fly rounds caught significantly more *G. m. morsitans* than *G. pallidipes*, the opposite was true of stationary traps (Wilcoxon rank sum test: *W* = 95.5, *P* = 0.06895 for trap catches and *W* = 1, *P* = 0.9944 for flyround catches).

### Additional traps

Additional traps deployed in the study area provided results that corroborated the findings from the transect. Figure 4 summarizes the findings in the additional traps, as well as those of the transect traps. Tsetse flies are either absent or present at extremely low levels in all sites south of the game fence. In fact, only one fly (*G. m. morsitans*) was caught south of the fence. In terms of apparent density, the findings in the additional traps are also consistent with the results from the transect (both fly rounds and traps). In particular, the highest densities were detected at the foot of the escarpment (Rekomechi and Marongora sites).

### Tsetse infection

Dissection of 2092 flies yielded only one positive infection of the *T. brucei*-type, while a number of *T. vivax*-type and *T. congolense*-type infections were identified (85 and 46, respectively). Both sexes of *G. m. morsitans* and *G. pallidipes* were dissected. A mean infection rate of 6.31% was found (132/2092). Infection rates were higher in males of both tsetse fly species than in females (7.32 and 5.28%, respectively) although statistically insignificantly higher (*t*-test: *t*_(40)_ = 1.0482, *P* = 0.3008). The infection rates did not differ significantly between the transect traps/fly rounds and traps deployed at Rukomechi Research Station (*t*-test: *t*_(4)_ = 1.1485, *P* = 0.3147). Shapiro-Wilk normality test resulted in *P*-values of 0.901 for Rukomechi infection rate and 0.9824 for transect indicating that data followed a normal distribution. The results of tsetse examinations are summarized in Table 1.

**Table 1:** Tsetse trypanosome infections detected by fly dissection and microscopic examination [19]

Location	No. of flies	*T. vivax*-type		*T. congolense*-type		*T. brucei*-type		Total	
		*n*	%	*n*	%	*n*	%	*n*	%
Transect traps and fly rounds	92	4	4.35	3	3.26	0	0	7	7.61
Additional traps (Rukomechi)	2000	81	4.05	43	2.15	1	0.05	125	6.25
Total	2092	85	4.06	46	2.20	1	0.05	132	6.31

## Discussion

The entomological surveys presented in this paper provide insights into the geographical distribution of tsetse flies in the sleeping sickness region of Zimbabwe (Hurungwe District). The game fence marking the limit between the protected and the settled areas appears to mark the southern limit of the tsetse fly distribution. This land use discontinuity is also associated with an abrupt topographic and ecological transition, i.e. the escarpment of the Zambezi Valley. All entomological findings confirmed that *G. morsitans morsitans* and *G. pallidipes* are present at varying densities from the Zambezi Valley floor to the top of the escarpment. Proceeding further south across the highlands, the tsetse fly infested area stretches to the game fence that marks the beginning of the settled and intensely cultivated area. The settled area presently appears fundamentally unsuitable for tsetse flies, although occasional incursions or relic populations may occur, as demonstrated by the one fly (*G. m. morsitans*) captured south of the fence in one of the ‘additional traps’. The presence of tsetse flies in the protected areas is consistent with the reported occurrence of HAT cases, which seem to be mainly linked to a wildlife reservoir of *T. b. rhodesiense* [7, 10]. As documented elsewhere [23, 24], the geographical interface between protected and settled areas appears to be one of the main areas where vectors (tsetse), parasites (trypanosomes) and susceptible hosts (humans and livestock) come together.

In view of the tsetse fly control operations which were discontinued approximately 15 years ago, and the land cover changes reported to have occurred in the intervening time, further studies are suggested to explore how the interplay between tsetse fly control and ecosystem change in the recent past may have shaped the present observed distribution of tsetse.

Within the tsetse fly infested area, very large differences in fly densities were observed, with peaks in the areas adjacent to the escarpment of the Zambezi Valley. The factors affecting these differences are not yet fully understood, but they are likely to be related to macro- and micro-climatic patterns and the availability of game hosts [25, 26]. Fine scale differences in vegetation cover, driven, for example, by the pattern of the hydrological network, may be playing a key role. In particular, watering points near the escarpment may provide both hosts and favourable microhabitat for tsetse flies.

The study also confirmed how the efficiency of fly rounds and traps differs greatly between the two species. *G.m. morsitans* are more attracted to mobile baits (fly rounds) while *G. pallidipes* are more attracted to stationary baits (traps) [27, 28] The bimodal pattern of tsetse fly diurnal activity was also confirmed, with no significant difference detected between the morning and afternoon fly rounds [16].

Results of tsetse fly dissections revealed a very low level of infections of the *T. brucei*-type. This finding seems to be consistent with a HAT epidemiological situation that is generally considered to be low risk [29]. On the other hand, molecular analysis using PCR on 209 flies collected during the same transect surveys revealed a higher prevalence (14.8%) of *T. brucei* DNA, although none of these tested positive for *T. b. rhodesiense* DNA (Goodwin et al., unpublished). This is due to the detection of both mature and immature trypanosome infections in the mid-gut and salivary glands. The detailed results of this molecular analysis will be published elsewhere.

The generally positive epidemiological picture or low prevalence of the disease emerging from these entomological surveys must be interpreted in the light of a low capacity for HAT diagnosis, especially in the endemic zones. Human African trypanosomiasis infections contracted in Zimbabwe are diagnosed almost exclusively in Harare or abroad [8, 11], and it is reasonable to speculate that many cases may go undetected. Efforts must be made to bring capacities for sleeping sickness diagnosis closer to the endemic areas and therefore reduce the travel time to equipped health facilities [27, 30]. A strengthened capacity for HAT detection would enable a more realistic estimation of the burden of the disease, and it would also contribute to tackling it more effectively. Also, the transboundary nature of the tsetse and trypanosomiasis problem calls for coordinated multinational actions, which in this particular case could involve Zimbabwe, Zambia and Mozambique. In areas within Zimbabwe that are affected by HAT (e.g. in safari hunting areas,), collaborative programmes with the relevant institutions should be prioritised. One such joint programme is currently underway with National Parks and Wildlife management to control tsetse in Hurungwe safari area.

## Conclusions

The present study confirms the usefulness of collecting and analysing spatially-explicit information on African trypanosomiasis, including entomological and parasitological data. For the future, more systematic data management and mapping at the national level is believed to be crucial for an evidence-based decision making in field interventions.

Long-term, geo-referenced data assemblies such as the one envisaged by the national Atlas of tsetse and AAT in Zimbabwe will be a prerequisite for investigating some of the epidemiological dynamics discussed in this paper (for example, the virtual disappearance of tsetse flies from areas south of the game fence). Elucidating the interplay between tsetse fly control and ecosystem and climatic changes in this area could help optimize tsetse control and elimination strategies in other zones in Zimbabwe and elsewhere.

Concerning the risk of sleeping sickness, the present study revealed an entomological situation that is consistent with the picture emerging from the reported HAT cases. In particular, infections seem to occur primarily in the protected, wildlife-rich areas with high tsetse fly density. Trypanosome infections of tsetse flies do not seem to indicate an alarming HAT risk situation. However, it must be noted that the relation between tsetse infection rates and HAT occurrence is a complex one, and a low HAT risk cannot be directly inferred from low infection rates in tsetse.

In the Hurungwe District, One Health approaches including targeted tsetse fly control have the potential to contribute to the reduction of both human and animal trypanosomiasis. In this context, the current study has considerable policy implications for Zimbabwe. In fact, while in the past control measures could target large swathes for tsetse eradication, only small and isolated patches of risk may be treated and targeted for elimination today. In these cases, the degree of isolation of these the target populations would need to be evaluated [31] to ensure sustainability. Targeted interventions can maximise the benefit-cost ratios in the present challenging economic context, and investment in integrated interventions against tsetse, HAT and AAT may hold the greatest potential. Importantly, while the overall long-term objective is the complete elimination of tsetse and trypanosomiasis from the entire country, because of resource constraints, the areas posing the greatest risk of HAT and AAT need to be prioritised.

## Abbreviations

AAT: Animal African trypanosomiasis
ANOVA: Analysis of variance
CASS: Centre for Applied Social Sciences
DDDAC: Dynamic Drivers for Disease in Africa Consortium
DFID: Department for International Development
EIDC: Environmental Information Data Centre
ESPA: Ecosystem Services for Poverty Alleviation
ESRC: Economic and Social Research Council
FAO: Food and Agriculture Organization of the United Nations
GPS: Global positioning system
HAT: Human African trypanosomiasis
NEPAD: New Partnership for Africa’s Development
NERC: Natural Environment Research Council
PAAT: Programme against African trypanosomosis
WHO: World Health Organization
WHO/TDR: World Health Organization/ Tropical Disease Research
